# A hands-free stool sampling system for monitoring intestinal health and disease

**DOI:** 10.1038/s41598-022-14803-9

**Published:** 2022-06-27

**Authors:** Sonia Grego, Claire M. Welling, Graham H. Miller, Peter F. Coggan, Katelyn L. Sellgren, Brian T. Hawkins, Geoffrey S. Ginsburg, Jose R. Ruiz, Deborah A. Fisher, Brian R. Stoner

**Affiliations:** 1 Electrical and Computer Engineering, Center for Water, Sanitation, Hygiene and Infectious Disease (WaSH-AID), Duke University, Durham, NC USA; 2 Duke Center for Applied Genomics and Precision Medicine, School of Medicine, Duke University, Durham, NC USA; 3 Division of Gastroenterology, School of Medicine, Duke University, Durham, NC USA

**Keywords:** Gastrointestinal diseases, Biomedical engineering, Gastroenterology, Biochemical assays

## Abstract

Analysis of stool offers simple, non-invasive monitoring for many gastrointestinal (GI) diseases and access to the gut microbiome, however adherence to stool sampling protocols remains a major challenge because of the prevalent dislike of handling one’s feces. We present a technology that enables individual stool specimen collection from toilet wastewater for fecal protein and molecular assay. Human stool specimens and a benchtop test platform integrated with a commercial toilet were used to demonstrate reliable specimen collection over a wide range of stool consistencies by solid/liquid separation followed by spray-erosion. The obtained fecal suspensions were used to perform occult blood tests for GI cancer screening and for microbiome 16S rRNA analysis. Using occult blood home test kits, we found overall 90% agreement with standard sampling, 96% sensitivity and 86% specificity. Microbiome analysis revealed no significant difference in within-sample species diversity compared to standard sampling and specimen cross-contamination was below the detection limit of the assay. Furthermore, we report on the use of an analogue turbidity sensor to assess in real time loose stools for tracking of diarrhea. Implementation of this technology in residential settings will improve the quality of GI healthcare by facilitating increased adherence to routine stool monitoring.

## Introduction

Noninvasive individual health monitoring conducted by smartphones, wearables and home sensors offers the advantages of objective and more frequent data collection, the convenience of remote monitoring, and holds promise for integration of this data into meaningful clinical assessments of disease progression and treatment response^1,2^. There is growing interest in remote health monitoring in gastrointestinal (GI) medicine for widely prevalent conditions such as functional GI diseases, autoimmune diseases, and colorectal cancer^3,4^. These conditions are estimated to affect as many as 1 in 10 people globally and significantly impact quality of life, ability to work, as well as healthcare costs^5–8^.

Traditionally underutilized, stool is an attractive biological specimen for remote GI disease monitoring because it can be collected non-invasively and longitudinally. Less invasive and costly than a colonoscopy, stool-based biochemical analysis allows for detection of many acute and chronic GI conditions such as GI cancer^9,10^, enteric infections such as *Clostridium difficile*^11^, response to treatment in inflammatory bowel disease^12,13^, and gluten consumption by celiac disease patients^14^. In addition to diagnosis of disease, access to fecal specimens coupled with molecular analysis and sequencing enables analysis of the gut microbiome, and pursuit of novel understanding of the etiology of disease and of novel therapeutic approaches in a number of intestinal and extra-intestinal conditions^15–18^. Time-dense stool analysis for the composition of the microbiota could provide an ability to discern the effect of pathophysiology from environmental factors such as dietary habits, drug treatments and intestinal motility^19^.

Despite being non-invasive and effective, implementation of stool-based analysis is severely limited in practice: patients are rarely able to produce a stool specimen during the clinical encounter^20,22–27^ and a number of studies from different geographies have found low uptake and adherence to GI disease surveillance based on regular stool testing^20–24^. Studies exploring barriers to surveillance by fecal tests have found that blood testing is preferred to fecal testing and suggest that only patients with active disease are likely to comply with a stool test^20,25^. Surveillance studies carried out when patients are stable consistently found adherence of only 30% for completing four or more repeated fecal tests^21,22,26^. Survey studies have revealed that a major reason for poor compliance on repeated fecal screening was avoidance/forgetfulness^27,28^ and over 60% of respondents stated that the reason for low acceptability of fecal tests was disgust/embarrassment in stool collection^23,25,29^.

Approaches to better leverage stool-based data and integrate stool analysis with routine use of the toilet have been recently proposed^30,31^. Emerging efforts by academia and toilet manufacturers for excreta monitoring within and around a toilet have focused on analysis of urine^31–37^.

Accessing fecal specimens is technically challenging due to the extremely sticky nature of the material and its heterogeneity^38,39^. Image capture of stool in the toilet bowl, either by the user^40^ or without user intervention by a camera in the toilet seat^31^, have been proposed to track stool appearance characteristics since significant disparities have been reported between patient assessment and stool properties such as consistency, particularly for diarrhea^41–43^. However, there are major social taboos and privacy concerns associated with operating a camera in a bathroom and particularly in the toilet seat.

Here, we report on an approach to isolating human feces just downstream from a flush toilet specifically designed for sample collection and analysis. Wastewater has been used for many years for molecular analysis-based surveillance of infectious disease at the community level for polio^44^, and since 2020, it has been extensively used for detection of SARS-CoV-2 to assess prevalence of COVID-19 infection at the community level^45–51^. In our approach, we have sought to improve the resolution of wastewater-based analysis to the level of a single toilet and individual stool being flushed through it. The purpose of this technology is to make the stool sampling process automatic and seamless with a daily routine for improved adherence to a testing regimen that enables better disease management and improved outcomes.

This work presents a proof-of-concept demonstration of hands-free human stool collection for continuous health monitoring. The approach separates stool from wastewater after the toilet is flushed for the purpose of analysis. A method based on buffer spray-erosion is used to collect a small portion of the stool for biochemical analysis in a manner that is amenable to automation and manages issues of cross-contamination. Two types of physiologically relevant data, occult blood measurements and 16S rRNA microbiome analysis, were conducted on fecal specimens obtained from our prototype system. The analytical integrity of the results was assessed by conducting pair-wise comparison against stool specimens sampled conventionally. For the clinically relevant cases of watery and loose stool, we demonstrate an inline analog turbidity sensor with the ability to discriminate loose stools from other types of wastewater for tracking of diarrhea.

## Results

### Design criteria

A foundational design criterion for our approach is to seamlessly integrate stool analysis with the routine use of the toilet without requiring any action nor habit change in users nor engendering discomfort. This criterion minimizes barriers to adoption and improves likelihood of long-term use for longitudinal data collection. We achieved this objective by leaving the toilet bowl appearance and function unchanged, and by operating in the plumbing where sampling and analysis occurs outside the purview of the user, after the specimen leaves the bowl.

Another key requirement is the ability to sample the stool in a manner that is independent from the toilet user and amenable to automation.

To be valuable as a health monitoring tool, the system must be able to detect the whole clinical range of stool consistencies from constipation to loose stools. A widely used standard medical diagnostic tool for categorizing adult stool based on its physical appearance is the Bristol Stool Form Scale (BSFS)^38,39^, featuring 7 types ranging from BSFS 1 (separate hard lumps, like nuts) to BSFS 7 (watery, no solid pieces).

### Design and operation

**Figure 1:**
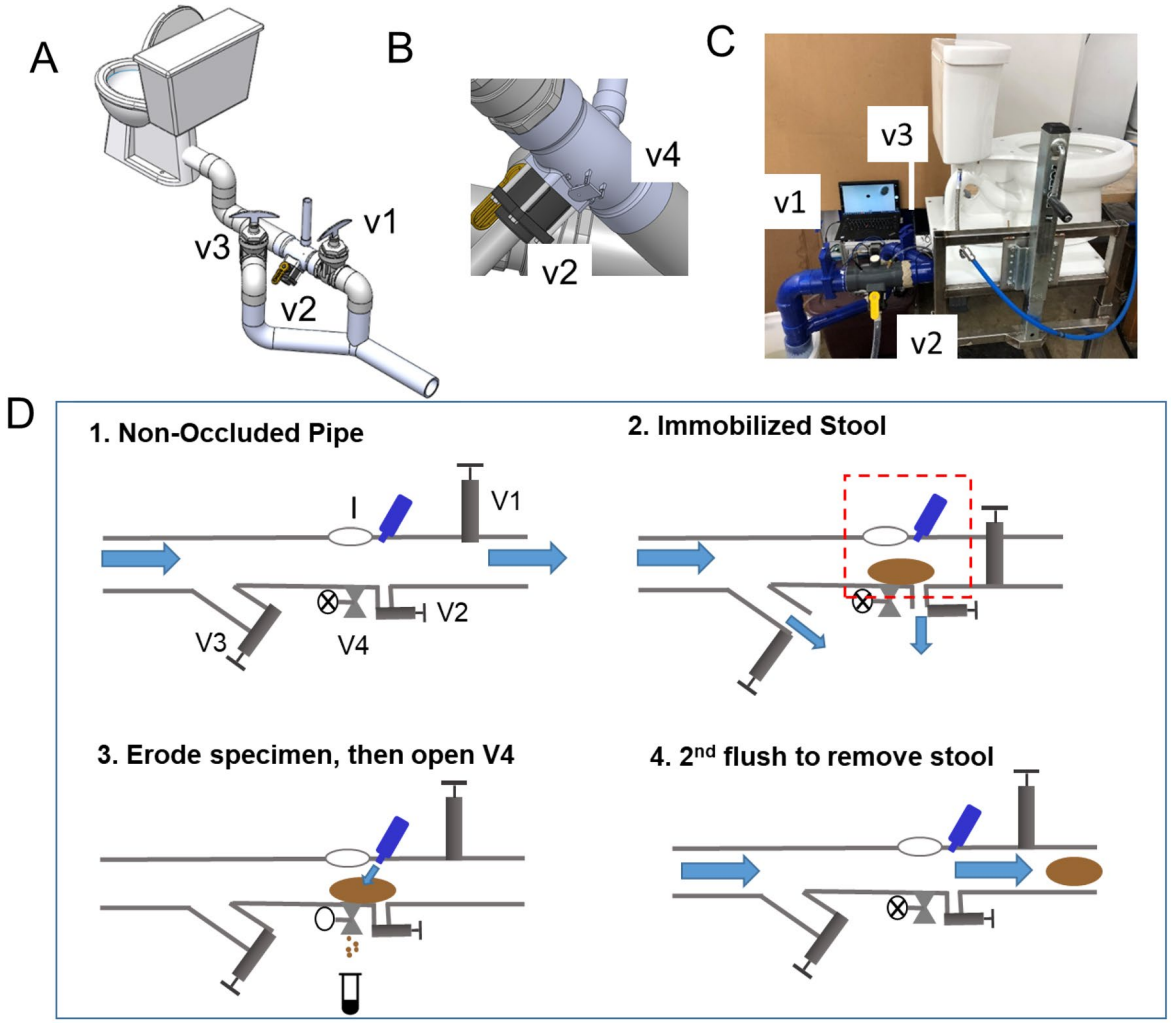
(**A**) An illustration of the prototype connected to a rear exit toilet. (**B**) Close-up view of the bottom part of the prototype shown in (**A**). (**C**) Picture of the prototype connected to a bottom exit toilet with endoscope connected to a laptop for image collection. (**D**) Operating principle of the system including 4 valves, V1 through V4, and I = inspection and venting port. 1. The valve position for regular operation of the toilet, blue arrow indicates water flush direction from toilet to sewer line. 2. The stool is immobilized in the stool area (red dashed rectangle). 3. A spray-jet of buffer erodes specimen collected by gravity through V4. 4. The stool is flushed to sewer.

**Figure 2:**
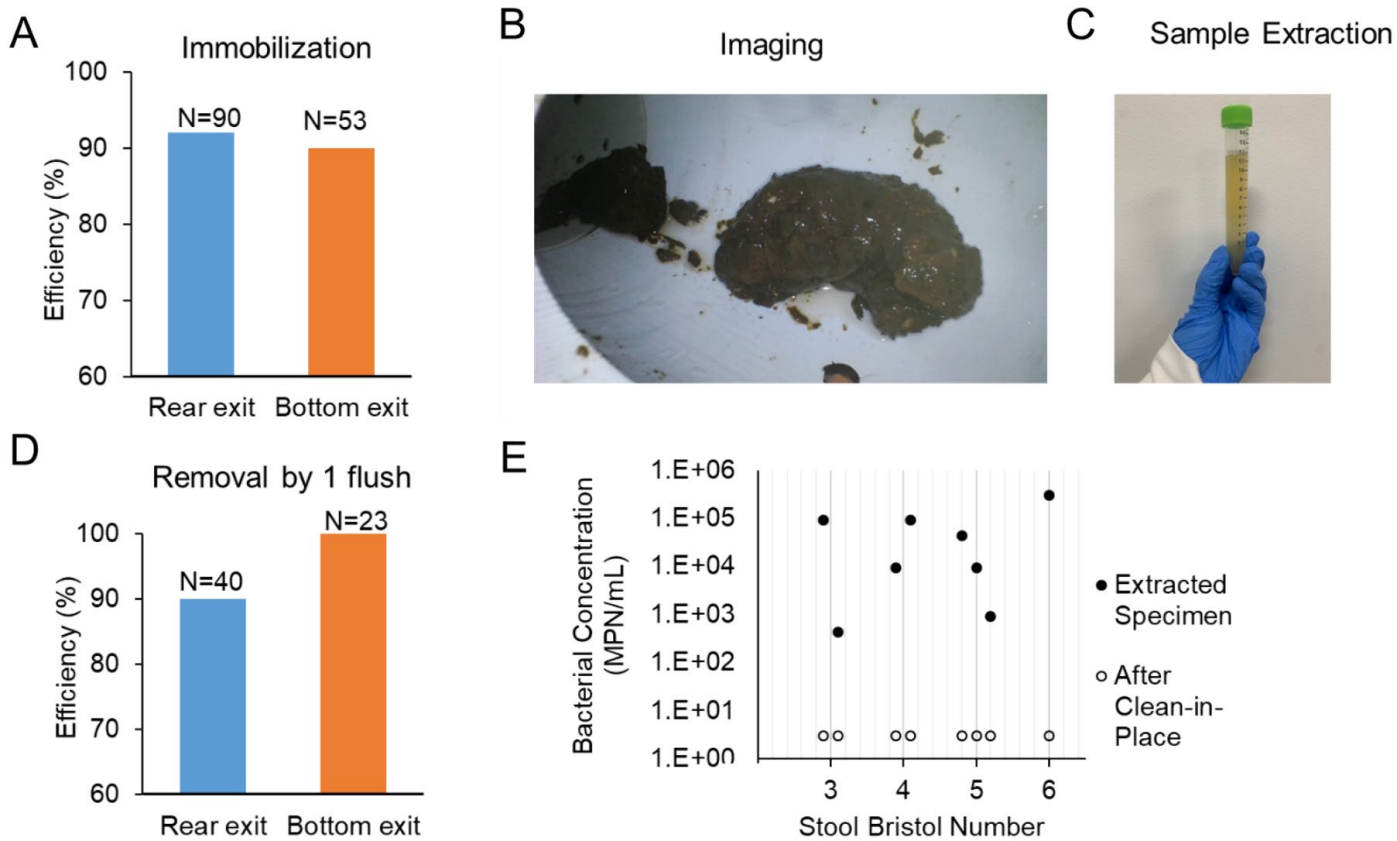
(**A**) Immobilization efficiency measured with formed surrogates and human stools in rear exit and bottom exit toilets. (**B**) In situ image of immobilized human stool in the prototype. (**C**) Image of spray-eroded extracted fecal specimen. (**D**) Efficiency of stool removal from the immobilization region with 1 toilet flush. (**E**) Bacterial enumeration by most probable number (MPN) for stool specimens of different consistencies (BSFS type) for specimen extraction and after the stool was removed and clean-in-place procedure implemented.

A standard flush toilet consists of a bowl that disposes of human excreta by using water to flush it through a drainpipe to another location for disposal. Critical to this design is a U-shaped section of outlet pipe that holds water—termed a P-trap or S-trap—that acts as a seal and prevents noxious gases from rising up through the toilet into the bathroom. Our design captures feces using a device integrated into the plumbing at the outlet of the toilet after the trap (Fig. 1) to preserve the odor sealing properties of the toilet. Reversible immobilization of the whole stool was achieved by solid/liquid separation and was demonstrated by flushing stool into a benchtop test bed (Fig. 1A–C). Immobilization was achieved using a combination of flow transport forces and occlusion by a gate valve V1 (Fig. 1D). When V1 was closed, inertia transported solid waste into a 3D printed custom designed component occluded by V1; water drained through valve V2 and through valve V3 located on a diversion pipe. The stool was immobilized near valve V2 and was not typically in contact with the occluding valve V1. The majority of the water drained through valve V3, ensuring the bowl was emptied with a similar speed as with a regular flush. We found that stool was immobilized in the region near valve V2 and aligned with the sampling port for 90% or more of the tests (Fig. 2A) in both types of toilets (90 flushes for the rear exit toilet and 40 for the bottom exit toilet); in the remainder of the cases the stool was typically immobilized between valve V2 and valve V1. Once stool was immobilized and the water drained, a stool image was collected through a port using a waterproof inspection camera (Depstech endoscope with 6 LED illumination) (Fig. 2B). Such images of individual stool are well suited for accurate determination of stool morphology by machine learning, as some of these investigators have previously reported^52^.

A specimen was extracted using a high-pressure liquid stream to erode and dissolve a portion of the immobilized stool (Fig. 2C). The buffer-eroded liquid specimen was collected by gravity through a zero dead-leg valve V4 (details in section “Specimen extraction”) (Fig. 1D, step 3).

As a last step of the operation, the remainder of the stool was disposed of to the sewer through a second flush while valve V1 occlusion was removed and all other valves were closed. Removal was achieved with a single flush in 90–100% of tests (Fig. 2D).

A major concern of sampling a sticky material such as feces is to ensure minimal cross contamination between specimens. Our design included a zero dead volume sampling valve for the purpose of eliminating stagnant volume or protruding surfaces. We also made use of the availability of tap water in the toilet operation to rinse surfaces. To determine the extent of specimen-to-specimen contamination upon stool immobilization, we measured fecal coliform bacteria, which are naturally present in stool.

Fecal bacteria were quantified using a most probable number (MPN) method on samples from a 3D-printed ABS device. The MPN assay was conducted on extracted stool specimens and samples from blank flushes following the stool disposal flush. The bacterial concentration was high in the specimen samples, as expected (> 10^4^ MPN/mL) (Fig. 2E). The sample collected after the disposal flush contained a much-reduced bacterial content, from 1 to 3 log reduction, likely associated by how “clean” the sample was removed. The clean-in-place procedure in this study consisted of a second flush. We tested specimens of different consistencies [value from BSFS 3 (sausage shaped) to BSFS 6 (mushy)] and for these tests, this flushing was adequate for the extracted volume to result in bacterial content below the limit of detection of the assay (3 MPN/mL).

### Hands-free stool sampling

**Figure 3:**
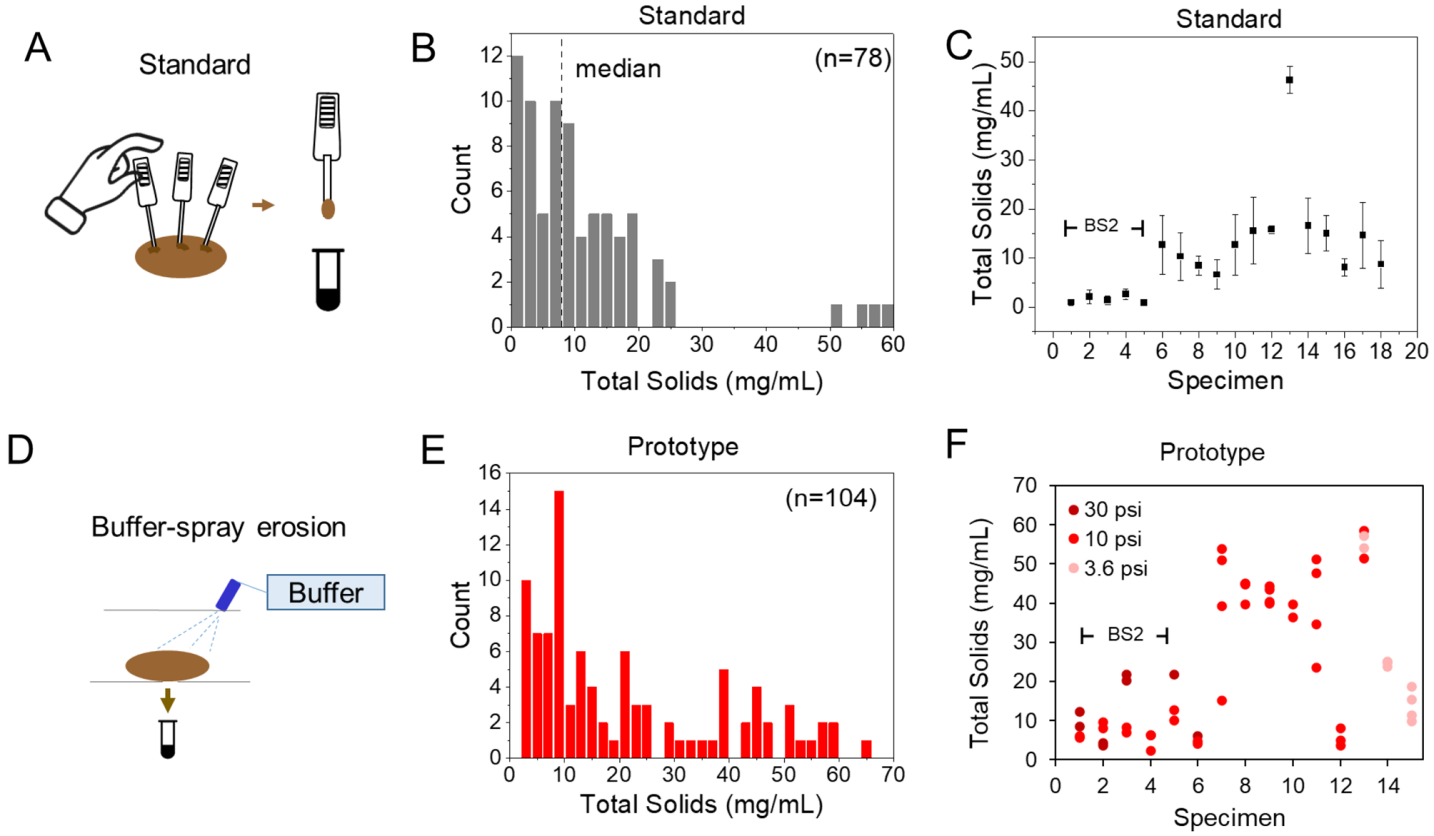
Specimen sampling study. Standard sampling: (**A**) Procedure illustration from the manufacturer. (**B**) Distribution of total wet solids with median value of 8 mg/mL. (**C**) Average and standard deviation of total solids from individual specimens, n = 3 to 5 for each specimen. BS2 = Bristol Stool Form Scale 2 indicates lumpy, hard consistency stool. Prototype sampling (D): Schematic. (**E**) Distribution of total wet solids collected from the prototype. (**F**) Total solids extracted from individual specimens using 5 s spraying time at the indicated pressure.

Standard sampling for fecal assays requires a person inserting a grooved stick or spatula in multiple regions of the stool and immediately diluting the specimen in buffer (Fig. 3). This process is challenging to automate within a wastewater plumbing environment because it requires a new stick for each measurement to avoid cross contamination.

We propose a novel sampling approach that is readily amenable to implementation in plumbing and automation and that is informed by the specific characteristics of feces. The water content of formed feces is on average 75%^53^, which is high as opposed to typical paste material. For example, the soybean surrogate used in these tests was measured at 54% water content, while the average water content for watery stools in cases of diarrhea is 88%^54^. The high water content of stool suggested that adding a small amount of liquid was sufficient to transform the solid stool into a liquid that is easier to manipulate and less sticky.

The fecal specimen extraction in our design relied on a spray jet of buffer to erode the stool and on collection of the liquefied sample by gravity through a valved opening. The advantages of this approach are multifold: no mechanical parts inside the pipe, readily amenable to automation (of the spray and valving) and ease of cleaning. We reasoned that this approach would yield a specimen suitable for biochemical analysis since dilution in buffer is the first step of most fecal assays.

Fecal assays require a small amount of stool (~ tenths of mg) and are often tolerant of a range of values. The wet total solids content in mg/mL is a measure of the dilution of the solid amount in buffer. Molecular tests for detection of pathogens in stool recommend a mass of 50–200 mg (see SI) resulting in 20–100 mg/mL wet solids while tests for more abundant species like microbiome require a vague “small” quantity of stool. To determine the stool amount for a point-of-care (POC) assay, we measured the amount of solid collected by the stick in a commercial occult blood at-home test (Fig. 3A). Figure 3B shows the results of solid content obtained using standard sampling over 18 stools and illustrates a broad range of values, with median 8 mg/mL, average 11 mg/mL and standard deviation of 12 mg/mL. The heterogeneity of stool form (BSFS 2–6 were measured) accounts for some of this variability; however, Fig. 3C shows that replicate measurements over the same stool specimens resulted in a wide variation (CoV between 0.2 and 0.7). The performance of our proposed extraction approach (Fig. 3D) was evaluated using a custom set-up with a tunable flowrate pump and connected to a precision nozzle.

We explored the amount of solid to be extracted by spray erosion. Measurements were conducted at multiple flowrates (between 0.4 and 1.1 L/min, corresponding to pressures between 3.6 and 30 psi, according to the nozzle specifications) and different spray times (2 s, 5 s, 10 s). We collected spray-eroded specimens in vials and determined total solids by drying as described in the methods.

Figure 3E shows that spray erosion enabled us to collect a wide range of wet solids from a few mg/mL to 60 mg/mL, with the histogram showing a broad range fully covering the range of the standard sampling by using different flowrate and spray times. To ensure this approach enabled collection of adequate fecal specimen solutions from a hard stool (BSFS 2), we showed that 10 psi produces values of 2–5 mg/mL, comparable to the standard method. By increasing to 30 psi we obtained over 20 mg/mL solids. No hard lump BSFS 1 stools were available for this study, but this data and the ability to increase pressure suggests that spray erosion can be tuned to obtain specimens from the whole range of stool consistency. For softer stool, a pressure of 3.6 psi was best suited to obtain a range of 10–20 mg/mL comparable to the 8 mg/mL median concentration obtained for standard sampling (Fig. 3F).

### Stool protein biomarker measurement

To demonstrate the feasibility of measuring a GI disease protein biomarker from stool specimens obtained from the prototype with spray erosion, we selected the well-established lateral flow assay (LFA) Fecal Immunochemical Test (FIT) to measure occult blood. The Fecal Immunochemical Test (FIT) for occult blood relies on detection of human hemoglobin and does not require any restrictions of food or medication intake. The FIT test features good sensitivity and excellent specificity for GI cancer^55^ and it is recommended worldwide and by the American Cancer Society as an annual screening for people beginning at 45 years of age who are at regular risk of GI cancer^9,10,56^.

**Figure 4:**
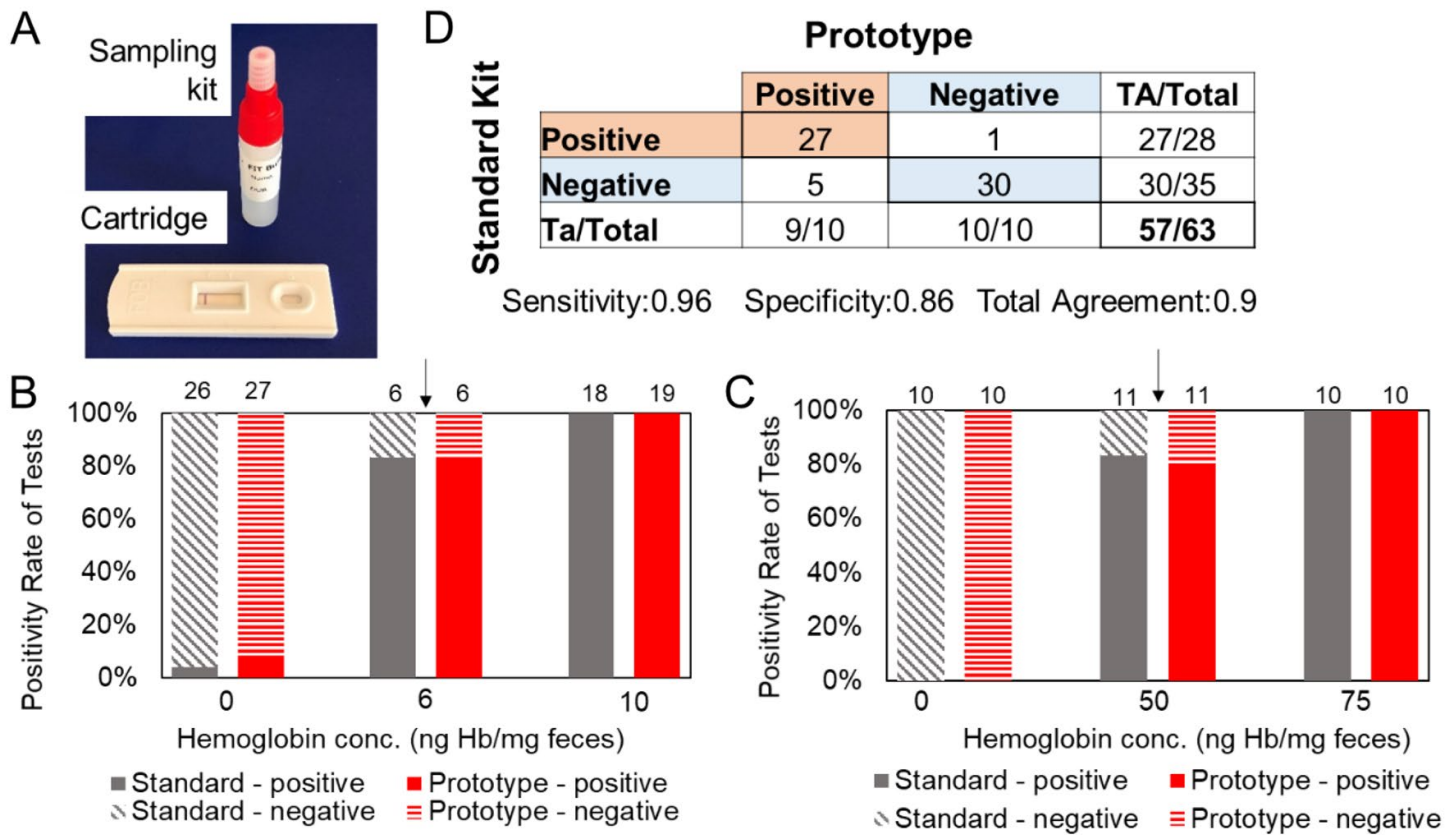
Effect of standard and prototype sampling on occult blood at-home test. (**A**) Picture of at-home kit (Pinnacle), including a tube with buffer, a screw cap and grooved stick for sample collection, and a cartridge for reading results. (**B**,**C**) The positivity rate (percentage of positive tests) of the (**B**) Pinnacle brand and (**C**) Accutest brand for human stools spiked at varying hemoglobin (Hb) concentrations. Black arrow: nominal limit of detection of the test. Figures on the second horizontal axis are the number of replicates. (**D**) The agreement matrix for pair-wise comparison on the same specimens at Hb concentration above the cutoff (10 ng/mg for Pinnacle brand, 75 ng/mL for Accutest).

Healthy stools are negative for occult blood and can be spiked with human hemoglobin to obtain a positive readout as has been done in proficiency tests of these kits^57^. Numerous FDA CLIA-waved FIT lateral flow assay kits for at-home use are available with qualitative (positive/negative) output. Our study evaluated brands Pinnacle and Accutest^57^. Both tests feature a sampling kit and a cartridge with an immunochromatographic strip for reading (Fig. 4A). The sampling kit is a small vial, containing a few mL volume (3 mL for Pinnacle, 2.5 mL for Accutest) of buffer, with a cap that is attached to a grooved plastic stick for stool collection. After inserting the grooved stick in the stool for a prescribed number of times (6 times), the user returns the grooved stick to the vial and shakes to dissolve the stool in the buffer. The kit vial has a smaller cap to dispense a prescribed number of drops of stool solution to the cartridge. Within 5 min from solution application, the control line of the cartridge appears and, in the presence of hemoglobin (Hb), the test line color appears (Suppl. Fig. S1).

The Hb detection limit is set by the manufacturer and it is typically 50 ng/mL. The Hb detection limit expressed as ng Hb per mg of stool is reported as 6 ng/mg for Pinnacle and 50 ng/mg for Accutest.

We compared the nominal sensitivity of the kit by testing a range of Hb spiking concentration spanning a range above and up to the nominal threshold. Using PBS as the buffer for erosion, we obtained by spray erosion specimens with wet solid content ranging from 5 to 25 mg/mL and applied a volume equivalent to the drops (75 μL) to the cartridge (Suppl. Fig. S1). Figure 4B shows a sensitivity curve for standard sampling with 100% positive readings (n = 18) at 10 ng/mg Hb, a value above the nominal sensitivity cutoff, and 4% (n = 26) positive for unspiked specimens. The reading of the cartridge samples from the prototype was similar, with 100% positive readings above the limit of detection and 7% positive for unspiked specimens. A similar study was done on the Accutest assay using 100 μL suspension (Fig. 4C) to strengthen the case that prototype sampling achieved the same analytical detection concentration as standard sampling.

To determine sensitivity, specificity, and total agreement of the test results obtained by prototype sampling relative to the standard method, we conducted pair-wise comparison of sampling methods on the same specimens at a fixed Hb spike concentration. For the Pinnacle assay, sensitivity was 95%, specificity 83%, and a total of 38 out of 43 pairs were concordant (88%) (Fig. S2). Discordant test pairs were mostly due to prototype sampling being positive while the standard was negative. This was typically caused by a large solids concentration (60 mg/mL in two cases). For the Accutest assay (Fig. S3), 19 of 20 pairs were concordant (95%). Overall, we found 90% agreement with 96% sensitivity and 86% specificity (Fig. 4D).

Using the Pinnacle assay, we also collected proof-of-concept data suggesting no impact on assay performance by interferents such as toilet paper (covering stool during spray-erosion) and urine (stool bathed in dilute urine mimicking a toilet bowl for 60 s prior to erosion). We measured 100% agreement with truth (defined as positive/negative result for spiked/unspiked stool respectively) for n = 5 test in each condition for both toilet paper and urine.

### Microbiome analysis

The objective of this experiment was to demonstrate the feasibility of microbiome analysis on specimens collected from our system. Stool specimens were obtained and sampled with a spatula for standard processing. Then, the same stool was placed in the toilet bowl, flushed and samples were collected by spray erosion. The study used 4 stools, S1, S2, S3, S4. When S4 stool was placed in the toilet bowl, 500 mL urine was also added for one minute prior to flushing to explore the possible contamination effects from urine, thereby we denote the specimen as S4u. S1 was measured in 6 biological replicates, S2, S3, S4u in triplicates for both sampling approaches. Six additional S1 replicates were centrifuged and the concentrated sludge used for analysis. A total of n = 36 samples underwent 16S rRNA microbiome analysis.

Extracted DNA concentration from the stool-sampling portal was found to be abundant (64 ng/μL as compared to 269 ng/μL for conventional sample) and more than adequate for 16S ribosomal RNA gene sequencing. All 36 samples submitted met the minimum criteria for analysis. The analysis of the relative abundance of bacterial taxonomic groups by order showed Clostridiales and Bacteroidales as the first and second most abundant, as expected in healthy subjects^58^ (Suppl. Fig. S4).

We quantified the microbial diversity within sample (*α* diversity) for standard sampling and our design sampling, and the difference between samples, i.e. between sampling approaches (*β* diversity).

**Figure 5:**
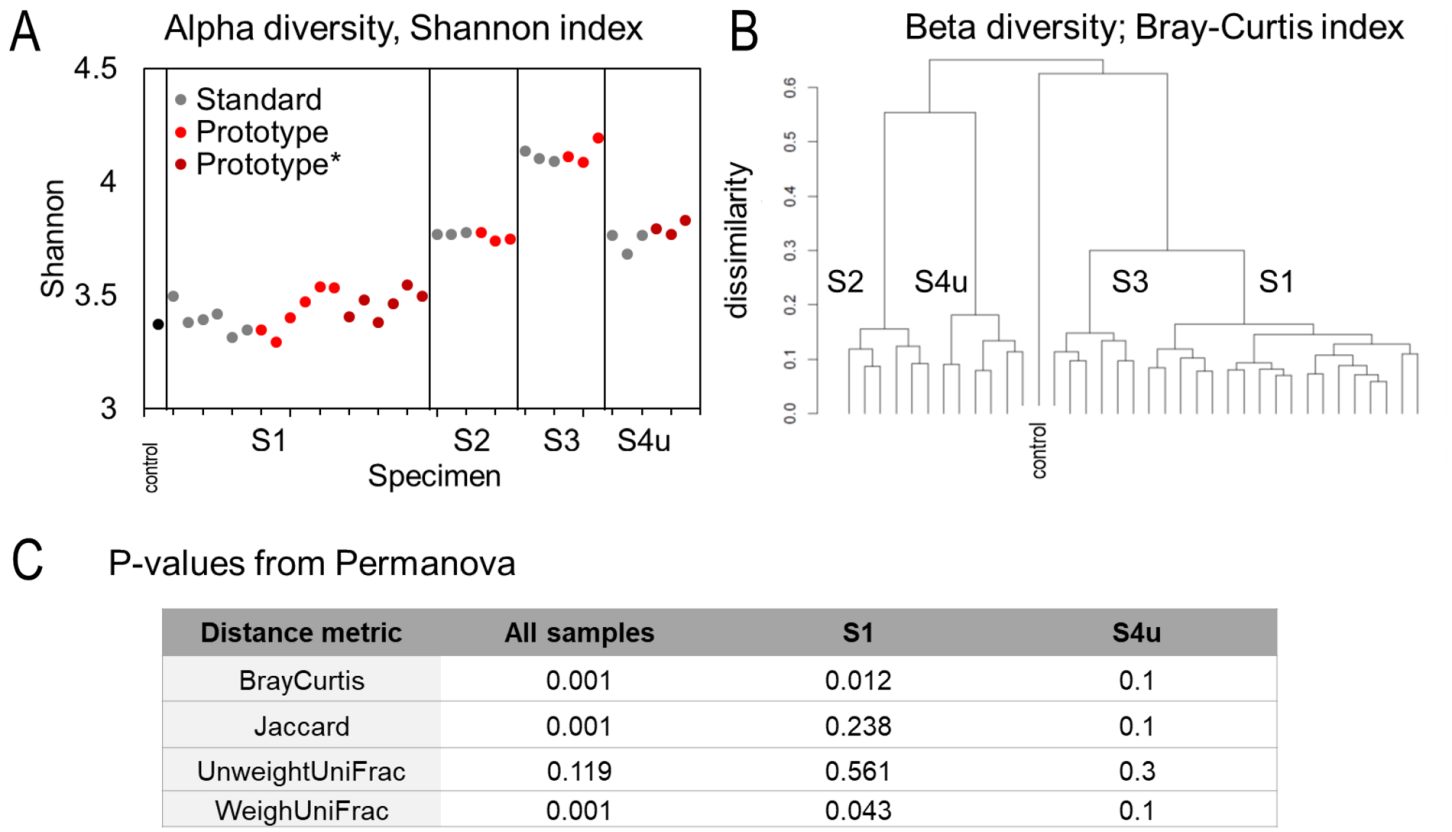
Microbiome analysis result of n = 36 fecal specimens from stool S1, S2, S3, and s4u. (**A**) Within-sample microbial species diversity (Alpha diversity, Shannon index) by stool and by sampling approach. * denotes sample centrifuged prior to nucleic acid extraction. (**B**) Hierarchical clustering using beta diversity index showing taxonomical distance: the clustering is around the stools not around the sampling approach. (**C**) P-value from permanova analysis of beta diversity metric comparing sampling methods (standard vs prototype) for test groups of interest: all stool samples (controlling for stools), stool 1 and stool 4u. P < 0.05 is statistically different.

Within-sample species diversity (*α* diversity, Shannon diversity index) did not significantly change across sampling approach (Fig. 5A). Alpha diversity analyses were performed using a linear mixed effects model with sampling approach as a fixed effect and stool as a random effect; p values were not significant, included for S4u, the specimen flushed with urine.

Beta diversity was calculated for each pair of specimens for multiple metrics (namely Bray–Curtis, Jaccard, UniFrac, and Weighted un-normalized UniFrac). Hierarchical clustering was performed using beta diversity metrics. A tree diagram illustration of the Bray Curtis beta diversity index in Fig. 5B shows the data clustering around specimen not sampling approach. These data illustrate that inter-individual variation of microbiome, a known feature of the microbiome of healthy individuals^59^ is a bigger factor than sampling approach.

A permutational multivariate analysis of variance (Permanova) of beta diversity was used to compare the two sampling approaches and test the null hypothesis.

The results were mixed depending on the metrics and the stool (Fig. 5C), with p values below p = 0.05 considered significant, indicating a difference introduced by sampling approach. We believe that the buffer type used in the spray-erosion sampling may introduce effects, since storage in different buffer types has been reported to influence fecal microbiome profiles^60^. Interestingly, for stool S4u, all four indices resulted in p = 0.1 or higher in the permanova analysis, supporting our hypothesis that urine contamination is a limited concern. In our design the contact time between urine and stool is limited in time as opposed to urine contaminating specimens collected with a stool collection kit.

### Detection of diarrhea

The stool sampling method based on solid/liquid separation is suitable for a wide range of stool consistencies. One limitation is that it cannot be utilized for completely watery stools (i.e., BSFS 7); however, several physiological conditions result in loose or watery stools, and the occurrence of such stools is itself an important GI biomarker. Specifically, diarrhea is clinically defined as the passage of 3 loose stools in a 24-h period. In conventional practice, this diagnosis generally relies on self-reporting and thus suffers from limited accuracy that impacts treatment. Here, we propose a non-invasive method for monitoring diarrhea that relies on an analog sensor in line with toilet effluent plumbing and is compatible with the configuration described in Fig. 1.

**Figure 6:**
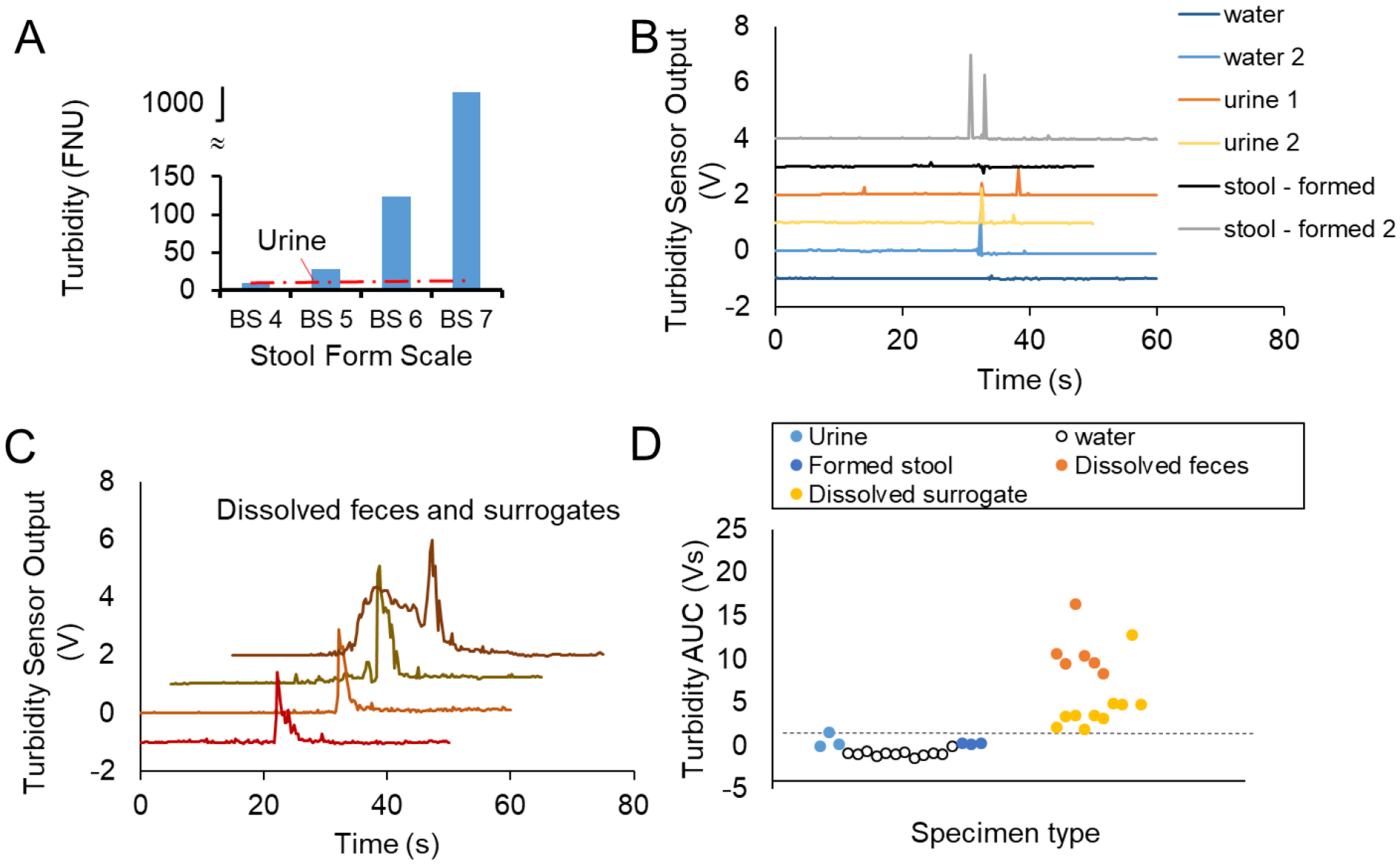
(**A**) Static Turbidity meter readings from solution containing stools of different form as defined by the BSFS measured after t = 1 min of immersion. (**B**) Sensor output recorded over toilet flushes (at t = 30 s) for water only and urine and formed stool. (**C**) Sensor output recorded for dissolved specimens flushed at predetermined time corresponding to peak (curves are shifted over both vertical and horizontal axis for clarity). (**D**) Cluster plot of area-under-curve of sensor output by specimen type, datapoints are spaced for clarity. Dotted line represents a proposed threshold of 2 Vs separating dissolved specimen from other wastewater types.

We found that nephelometric turbidity, a light-scattering-based measurement of the suspended particles in solution, is a sensitive measure of the presence of dissolved stool in a water solution. In batch static (no flow) tests we mixed water, human urine, and feces (both formed and liquid) in concentrations emulating one toilet use, as described in the Methods. Formed stools in water resulted in turbidity values ranging from 10 to 120 FNU (formazin nephelometric unit), significantly different from water baseline (T = 0.7 FNU) and from urine added to water (T = 1.1 ± 0.3 FNU, n = 5). Stool completely dissolved in water, emulating a BSFS 7 watery stool, resulted in saturation of the turbidity reading > 1000 FNU (Fig. 6A).

We used a waterproof analog turbidity sensor (SEN0189) designed to monitor the haziness of water in home appliances to obtain a dynamic measurement of turbidity within wastewater plumbing. The sensor was calibrated against the Hach 2100Q turbidity meter (Suppl. Fig. S5) and found to be linear up to 30 mg/mL wet solid content, more than adequate to detect diarrhea in toilet wastewater. The effect of toilet paper on the turbidity sensor was found to be negligible. Using an amount of toilet paper informed by ASME standard 112.19.2 for Ceramic Plumbing Fixtures (in “Methods”), we performed paired measurements across different concentrations of stool surrogate (soybean paste) and/or under different conditions (stirred or settled), with and without toilet paper added. Toilet paper did not result in significantly different readings (p = 0.7334 for n = 12 paired measurements with and without toilet paper).This was not surprising since toilet paper disintegration time ranges from 10 min to hours^61^, and is facilitated by extensive turbulence in a sewer system, well beyond the small pipe exiting a toilet^62^.

We installed the turbidity probe in the benchtop setup of the rear exit toilet to evaluate its dynamic response during toilet flushing. The probe was installed in the wastewater pipe immersed in the water of the S-trap and the electronic adapter was outside of the pipe (Suppl Fig. S6). The sensor probe output was collected with the following protocol: 60 s recording, stool or urine poured in the bowl at t = 10 s, toilet flushed at t = 30 s. Figure 6B illustrates typical readout for water and formed stool: the turbulence created by a flush may result in a short noise spike during at the time of the flush, but the output is otherwise unchanged. The flushing of a turbid solution of either dissolved feces or surrogate created the signature of a peak of a few seconds wide at t = 30 s (Fig. 6C). Occasionally, the pouring of dissolved specimen in the bowl prior to flushing resulted in a turbidity sensor signal (top curve in Fig. 6C) indicating the dissolved specimen had crossed the S-trap and reached the sensor without a toilet flush. The Area-Under-Curve measured for all signals measured around the flush is plotted in Fig. 6D. Values for water, urine and formed stool are either zero or below 2 V-s; values measured for dissolved specimens are above 2 V-s indicating that the AUC metric can be used as a threshold to discriminate the presence of loose stool.

## Discussion

Aversion to handling one’s own stool is arguably the most significant barrier to harnessing health data from this physiological specimen^23,25,29^. This study demonstrated feasibility of stool collection from toilet effluent and suitability of the specimen for biochemical analysis.

Previously published work on toilet-related stool analysis used sensors or receptacles in the toilet bowl or seat for stool imaging and did not achieve the diagnostic specificity of biomolecular assays^31,34^. We report a configuration that achieves solid/liquid separation of a stool specimen in a specified region of the wastewater plumbing and stool sampling by spray-erosion. This configuration is advantageous because it minimizes mechanical parts inside the sewer conduit that may foul and fail, does not induce mechanical smearing of the specimen that may cause cross-contamination, and is amenable to automated control.

This stool sampling approach occurring after the use of a conventional toilet outside the purview of the user will remove significant barriers to stool specimen collection. We envision the stool sampling system installed in the home for longitudinal monitoring of intestinal health and disease. The stool analysis may be conducted on site, by POC devices measuring a biomarker appropriate for a diagnosed chronic condition, or by laboratory analysis for health screening and research purposes.

We demonstrated analytical integrity of the sampled fecal specimen using established clinical POC kit for occult blood for screening for GI cancer. We believe the approach can be applied to additional stool biomarkers for which frequent monitoring improves clinical outcomes. For example, the approach could be used to measure fecal calprotectin which is gaining widespread use in treatment decisions for Inflammatory Bowel Disease^63^. Stool-based POC assays can also be used to detect inadvertent gluten exposure in patients with celiac disease on gluten-free diets^14^.

The sampling approach of this study yielded specimen suitable for microbiome analysis. While limited in number of samples, our data suggest that 16S rRNA microbiome analysis can be readily carried out with spray-eroded samples and that the mixing of urine with stool in wastewater does not appear to cause a measurable effect. Further studies are needed to optimize buffer selection and implementing automated sealing of liquefied specimens for shipment to a laboratory.

We note that the two assays used in this study do not depend on the exact amount of feces analyzed, as is also the case of molecular-analysis based fecal pathogen screening. Future work will examine feasibility of quantitative fecal assays, such as metabolite analysis, by analyzing the solid pellet obtained from centrifuging a spray-eroded fecal specimen.

We also demonstrated a real-time sensing approach for loose stools in wastewater. The occurrence of loose/watery stool over time is clinically important to appropriately diagnose and treat diarrhea. Despite the fact that a visual assessment of the stool can be readily implemented after toilet use, tracking of bowel movement is burdensome and prone to inaccuracy and may delay and otherwise degrade therapeutic intervention, particularly in a common and chronic condition such as Irritable Bowel Syndrome^43^.

This study was a feasibility demonstration and has some limitations. The test system was twice as large a toilet and would not fit within the footprint of a standard bathroom (with 12″ distance between the floor drain and the wall per building code). Ongoing engineering efforts focus on making the system more compact so that it can be installed in a bathroom with no infrastructure modification, and on automation of operation of flow valves. This study was also limited to the use of healthy human stools collected outside of the toilet system, Future work will demonstrate the approach with clinical specimens and human subjects using the system. A major factor in toilet use in the western world, toilet paper, was not systematically evaluated in this study, although preliminary data suggests that toilet paper does not impact results in POC test and turbidity measurements.

Looking at the ultimate implementation of this technology as a home monitoring system, we anticipate that the cost will be affordable because the system does not require specialty materials or high-performance actuation, and essentially relies on pipe shapes and commercially available valves to regulate water flow from the existing cistern flush. We envision a system with a cost at the midrange of current toilet prices and whose purchase may ultimately be covered by medical insurance.

In conclusion, we have presented a configuration that enables individual stool specimen collection from toilet wastewater for biochemical assays. The data here presented support the further development of the approach to achieve seamless stool biomarker data collection for a higher standard of monitoring of intestinal health and disease.

## Methods

### Specimens

Feces and urine samples were collected from healthy anonymous volunteers according to a protocol approved by Duke University Health System IRB 0010-5536. All the study procedures were conducted in accordance with the declaration of Helsinki and Duke University relevant regulations and procedures, and informed consent was obtained from all participants.

Feces was collected in plastic toilet hats and stored at 4 °C and before any test, it was allowed to equilibrate at room temperature for 2 h. Urine was collected in sterile 500 mL bottles and stored at 4 °C until use. As surrogate for feces, experiments were conducted with cylinders of soybean paste^64^, both in latex casing and uncased, as prescribed in ASME 112.19.2 Ceramic Plumbing Fixtures testing standards. Amount used in testing with surrogates is 100, 150 and 200 g or two samples of 50 g. Human feces amount used in experiments is either 50 or 100 g, unless otherwise indicated (the median wet weight of stool in high income countries is 128 g^53^). The volume of urine passed each time by a normal adult typically ranges from 250–400 mL, with median values reported as 233 (1400/6) mL^53^.

### Prototype

The system was tested with commercial wash-down toilets of two types: a rear exit toilet (Saniflo 093) and a bottom exit toilet (Kohler Wellworth K-4198), with water efficient cistern (4.8 L flush). The volume of water in the toilet bowl was measured as 1.85 ± 0.17 L for the bottom exit toilet. A rear outlet P-trap connector (Signature Hardware) with rubber seal was installed at the 3″ exit of the toilets, and 3″ PVC pipes, elbows and connectors were used to connect to the custom design part. The stool immobilization and sampling pipe was drawn using SolidWorks and 3D printed in multiple versions in either PLLA and ABS material. It includes a gentle dip and sampling port opening located at the bottom to collect liquid by gravity. Valve 1 is a 3″ gate valve, V2 is 2″ gate valve; Valve 3 is a ball valve that mates to a connector included in the 3D-printed part to minimize dead volume. Valve 4 for sample extraction was a 3D printed swing-type closure with rubber stopper sized to ensure zero dead leg.

### Specimen extraction

The sample collection kit of an occult blood assay (Pinnacle Biolabs) was used to measure the stool mass used in standard sampling. The stool mass on the grooved stick from the kit was measured using an analytical balance. The balance scale was also used to measure the volume of buffer in the collection kit (3 mL and 2.5 mL). The wet solid content of the suspension prepared with the standard kit was calculated by ratio.

Spray erosion was achieved with a pump (12 V PowerFlo), powered by a tunable voltage power supply. Buffer was pumped through a ¼ inch nylon tubing and an adapter to a precision nozzle (Lechlert 632.404). The flow rate was measured in the range of 0.4–1.1 Liter per minute, and spray pressure was calculated from the nozzle manufacturer chart. Fecal specimens of 35 g each were used. The liquefied specimen was collected by gravity from the bottom sampling port in a 15 mL sterile centrifuge tube.

In order to compare results of spray-erosion with the standard sampling, the wet solid content of the eroded suspension was determined. First, dry solid mass content was measured according to the conventional total solids method (oven at 105 °C for at least 4 h). Given the definition of moisture content MC (%) = (wet weight − dry weight)/ wet weight; the wet solid weight was calculated using a nominal value MC of 75% for feces. Moisture content of feces ranges between 50 and 80% with a median value of 75% across multiple studies^53^. All the solid content results reported in this work are wet solid weight.

### MPN analysis

Bacteria were enumerated using a most probable number (MPN) method as previously described^65^. Samples were collected in sterile centrifuge tubes and were stored at 4 °C until plating. Serial dilutions (10^−1^–10^−8^) of each sample were made in triplicate in lysogeny broth in sterile 48-well cell culture plates. Samples were incubated at 37 °C for 48 h before being analyzed.

### Occult blood assay

Two assays detecting human hemoglobin (Hb) were used: 1. the Second Generation FIT by Pinnacle Biolabs, with nominal cutoff at 50 ng/mL Hb in buffer (equivalent to 6 μg Hb /g feces); and 2. Accutest^®^ iFOBT, by Jant Pharmacal Corporation with nominal cutoff at 50 ng/mL Hb in buffer or 50 μg Hb/g feces.

The kits were used per manufacturer instruction. The grooved stick was stabbed 6 times into the stool specimen, then the grooved stick was screwed to the buffer tube (3 mL Pinnacle, 2.5 mL Accutest), and manually shaken. A tip cover of the tube was removed to dispense (3 drops, Pinnacle measured as 75 μL, or 4 drops measured as 100 μL, Accutest) of suspension on the cartridge.

Human hemoglobin (H7379-1G, Sigma Aldrich) was used for spiking. A custom procedure was developed to spike whole stools while maintaining form. The process consists of manual folding for 50 times the whole stool mixed with spiking solution (100–500 μL of solution for 100 g of solid) using a spatula as shown in Supplementary Video S3.

#### Effect of urine

For this test to be reproducible fixed amounts of stool and urine were mixed in a container on the bench and then manually placed in the prototype for spray-erosion. The upper limit of urination volume of 500 mL was used. For practical reasons, the amounts were scaled down by a factor of 5. We combined 200 g/5 = 40 g feces, 500 mL/5 = 100 mL urine and 1.85/5 = 0.4 L water (representing bowl volume). After 1 min, the feces was collected with a slotted spoon and placed in the prototype for spray-erosion at 10 psi for approximately 5 s.

#### Effect of toilet paper on occult assay

For this test to be reproducible we combined fixed amounts of stool and toilet paper in a container and placed them in the prototype for spray-erosion. We collected the specimen with a grooved stick for the occult blood assay by standard method before adding the toilet paper. We used 40 g of stool and 6 sheets of toilet paper and combined with 400 mL of water to make the toilet paper completely moist. Using a slotted spoon, we collected the stool and toilet paper and placed it in the prototype ensuring the toilet paper was covering the stool. We conducted spray-erosion at 10 psi for approximately 5 s and collected the eroded liquid with no change of procedure.

### Microbiome analysis

Stool were sampled per instructions from the Duke Genomics and Computational Biology (GCB) Microbiome Shared Resource (MSR). A metal spatula reached the core of the stool and extracted ~ 250 mg sample. Six such collections were performed from separate regions of the stool, each reaching into the core of the stool. The extracted samples were placed in a 1.2 mL cryovial.

Then the same stool was placed in the toilet bowl and flushed, and sequential spray-eroded specimens were collected in a 15 mL sterile centrifuge tube. A portion of collected liquid samples was transferred to a 1.2 mL cryovial. For specimen S1, six replicates were centrifuged at 5000 RPM for 10 min and the supernatant discarded to provide a more concentrated sludge for analysis.

After storing overnight at − 20 °C, specimens were transferred on ice to GCBMSR where they were stored at − 80 °C until processed according to standard protocols.

Total microbial DNA was extracted using protocols standardized by the Human Microbiome Roadmap Initiative. DNA was isolated using the Qiagen PowerSoil ProDNA Isolation Kit. Extracted DNA concentration was measured using Nanodrop8000 spectrometer and found as abundant as 64 ng/μL for the processed sample and 269 ng/μL for the conventional sample.

Bacterial community composition in isolated DNA samples were characterized by amplification of the V4 variable region of the 16S rRNA gene by polymerase chain reaction using the forward primer 515 and reverse primer 806 16S rRNA following the Earth Microbiome Project protocol. The primers (515F and 806R) carry unique barcodes allowing for multiplexing and co-sequencing of hundreds of samples at a time. Equimolar 16S rRNA PCR products were quantified using Promega GloMax and pooled for sequencing. Sequencing was performed on an Illumina MiSeq sequencer with 250 base-pair paired-end sequencing runs (with up to 384 samples per lane sequencing runs). Raw reads were processed into Amplicon Sequence Variant (ASV) count tables via Qiime 2.0. Data underwent quality control by denoising and dereplicating the reads and by additional filtering. Taxonomy was assigned to ASVs using the q2‐feature‐classifier classify‐sklearn naïve Bayes taxonomy classifier against the SILVA 132 database. The negative extraction control was used to filter out potential contaminant ASVs using both prevalence and post-PCR DNA quantification thresholds via the Decontam package in R.

### Turbidity measurements

For static turbidity measurements, a calibrated turbidity meter Hach 2100Q (Hach, Colorado, USA) was used. A single toilet use was emulated using excreta physiological values of 130 g stool mass and 400 mL urine in 4.8 L cistern flush volume, resulting in 0.03 g/mL feces, 0.08 mL/mL urine in water vol/vol. We represented the use of toilet paper according to values of 4 balls of 6 single ply sheets required by ASME Standard 112.19.2.

For dynamic measurements, dissolved specimens were obtained from formed samples by diluting 1:1 by weight in water and mixing to obtain homogeneous suspension for replicate 100 mL test measurements.

An analog turbidity probe with cables and board (SEN0189 by DF Robot) was connected to an Arduino Uno board (DFRduino UNO v3, DF Robot). Arduino was used to develop the sensors code and Teraterm software to convert the recording into a .CSV file was used to log the turbidity sensor data using temporal resolution of 0.25 s.

### Statistical analysis

All data except the microbiome data were plotted in Microsoft Excel 2016. Turbidity data were analyzed with GraphPad Prism 9.1.2. Normality of differences between paired turbidity measurements was checked by the Anderson–Darling test, with a *p* value of < 0.05 considered statistically significant. Since the differences in measurements failed the normality test (p = 0.0006) a non-parametric test (Wilcoxon matched-pairs signed rank test) was used to determine if the differences between paired measurements were significant (p < 0.05).

## Supplementary Information

Supplementary Information.

Supplementary Video S1.

Supplementary Video S2.

Supplementary Video S3.

## Data Availability

All data associated with this study are present in the paper or the Supplementary Materials. All information and materials can be requested from the corresponding author.
