# Supplementary material for: A hands-free stool sampling system for monitoring intestinal health and disease
Source: Sci Rep. 2022 Jun 27;12:10859. doi: 10.1038/s41598-022-14803-9 (PMC9237014; doi:10.1038/s41598-022-14803-9)
Supplement: Supplementary file 1 — Supplementary Information. [file 41598_2022_14803_MOESM1_ESM.docx]

**SUPPLEMENTARY INFORMATION**

**A hands-free stool sampling system for monitoring intestinal health and disease**

Sonia Grego^1*^, Claire M. Welling^1^, Graham H. Miller^1^, Peter F. Coggan^1^, Katelyn L. Sellgren^1^, Brian T. Hawkins^1^, Geoffrey S. Ginsburg^2^, Jose R. Ruiz^3^, Deborah A. Fisher^3^, and Brian R. Stoner^1^

^1^ Electrical and Computer Engineering, Center for Water, Sanitation, Hygiene and Infectious Disease (WaSH-AID), Duke University, Durham, NC, United States

^2^ Duke Center for Applied Genomics & Precision Medicine, School of Medicine, Duke University, Durham, NC, United States

^3^ Division of Gastroenterology, School of Medicine, Duke University, Durham, NC, United States

**Table of Content**

**Supplementary methods**

**Figure S1**: illustration of the assay steps for standard and prototype sampling

**Figure S2:** Occult blood concordance matrix for brand 1

**Figure S3**: Occult blood concordance matrix for brand 2

**Figure S4:** Microbiome taxonomy distribution of specimen obtained by standard and this technology sampling

**Figure S5:** Turbidity sensor calibration

**Figure S6:** Pictures of the turbidity sensor and its installation in the toilet system

**Video S1:** Operation of the prototype after flushing of a formed specimen

**Video S2:** Examples for spray erosion on feces with different consistency.

**Video S3:** Spiking procedure illustrate with dye color and soybean surrogate

**Supplementary Methods**

**Stool Spiking method.**

Mixing the analyte with feces represents the closest approximation of uniform analyte distribution within a fecal sample. Spiking of fecal specimens is typically performed by homogenizing a small quantity (0.2 to 2 g)^1^. However, this is not applicable to this project since we aim to test stool samples of 50-100 g. Feces are thixotropic, meaning that their apparent viscosity decreases under shear stress (i.e., mixing), and the apparent viscosity of feces does not fully recover following shear^2^. In fact placing stool (or soybean past surrogate, that is also thixotropic^3^) in a low speed blender results in a liquid that can no longer be formed in a cylinder. Uniform spiking of an entire stool (~100 g) is not trivial. For example, in order to rigorously test the occult blood in stool, stool samples were “spiked” by collecting stools from clinically normal subjects after ingestion of autologous blood^4^. We observed that manual folding with a flat spatula for a large number of folds (as many as 50 times) enables uniform spiking of food dye with soybean surrogate (500 μl dye per gram solid) with no streaks per visual observation. Supplementary video S3 illustrates the procedure.

References

1. Barrows, G.H., et al., Immunochemical detection of human blood in feces. *American journal of clinical pathology* **69**(3), 342-346 (1978).

2. Woolley, S., R. Cottingham, J. Pocock, and C. Buckley, Shear rheological properties of fresh human faeces with different moisture content. *Water sa* **40**(2), 273-276 (2014).

3. Lee, C.-W., E.-S. Hwang, S.-Y. Lee, and Y.-R. Pyun, Rheological properties of doen-jang (Korea fermented soybean paste) suspensions. *Korean Journal of Food Science and Technology* **22**(2), 111-115 (1990).

4. Harewood, G.C., et al., Detection of occult upper gastrointestinal tract bleeding: performance differences in fecal occult blood tests. . *Mayo Clinic Proceedings* **77**(1), 23-28 (2002).

**Figure S1:** illustration of occult blood assay steps according to A. Standard Sampling (adapted from Accutest User instruction) and B. the proposed sampling method.

**
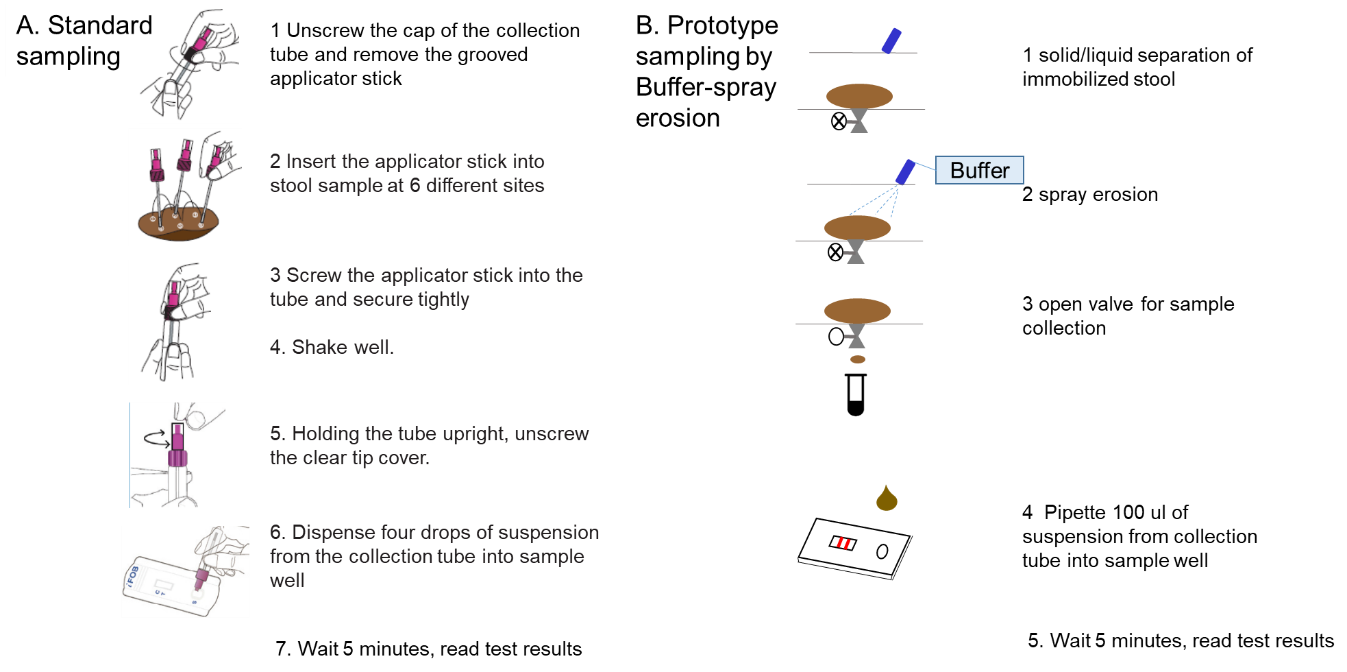
**

**Figure S2** Occult blood concordance matrix for Pinnacle assay and paired specimens sampled according to the standard method and in the prototype. Positive specimen were spiked at 10 ngHb /mg feces.

**
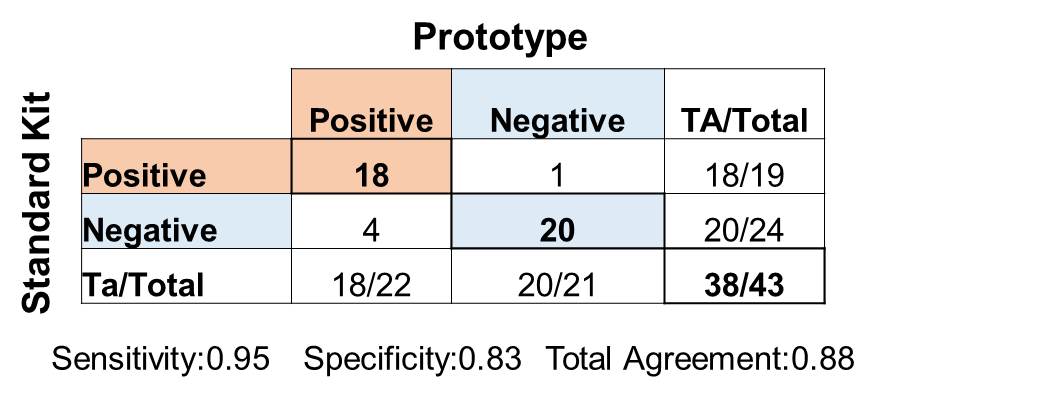
**

**Figure S3** Occult blood concordance matrix for Accutest assay and paired specimens sampled according to the standard method and in the prototype. Positive specimen were spiked at 75 ngHb /mg feces.

**
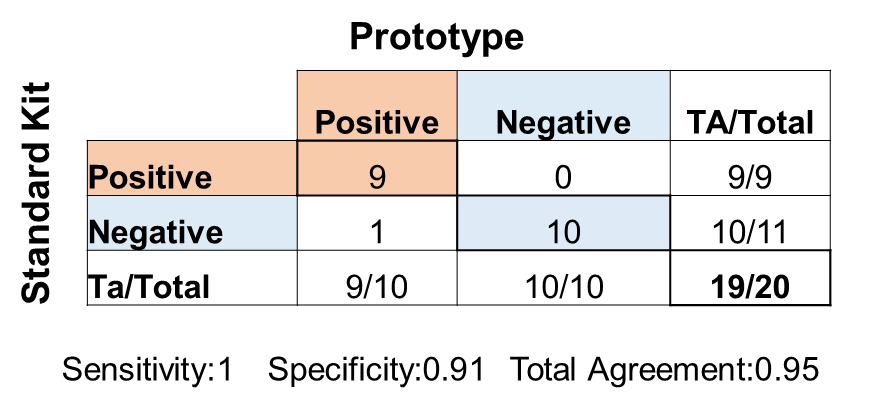
**

**Figure S4** Taxonomy distribution by bacterial order for specimens S1, S2, S3 and S4-u.

**
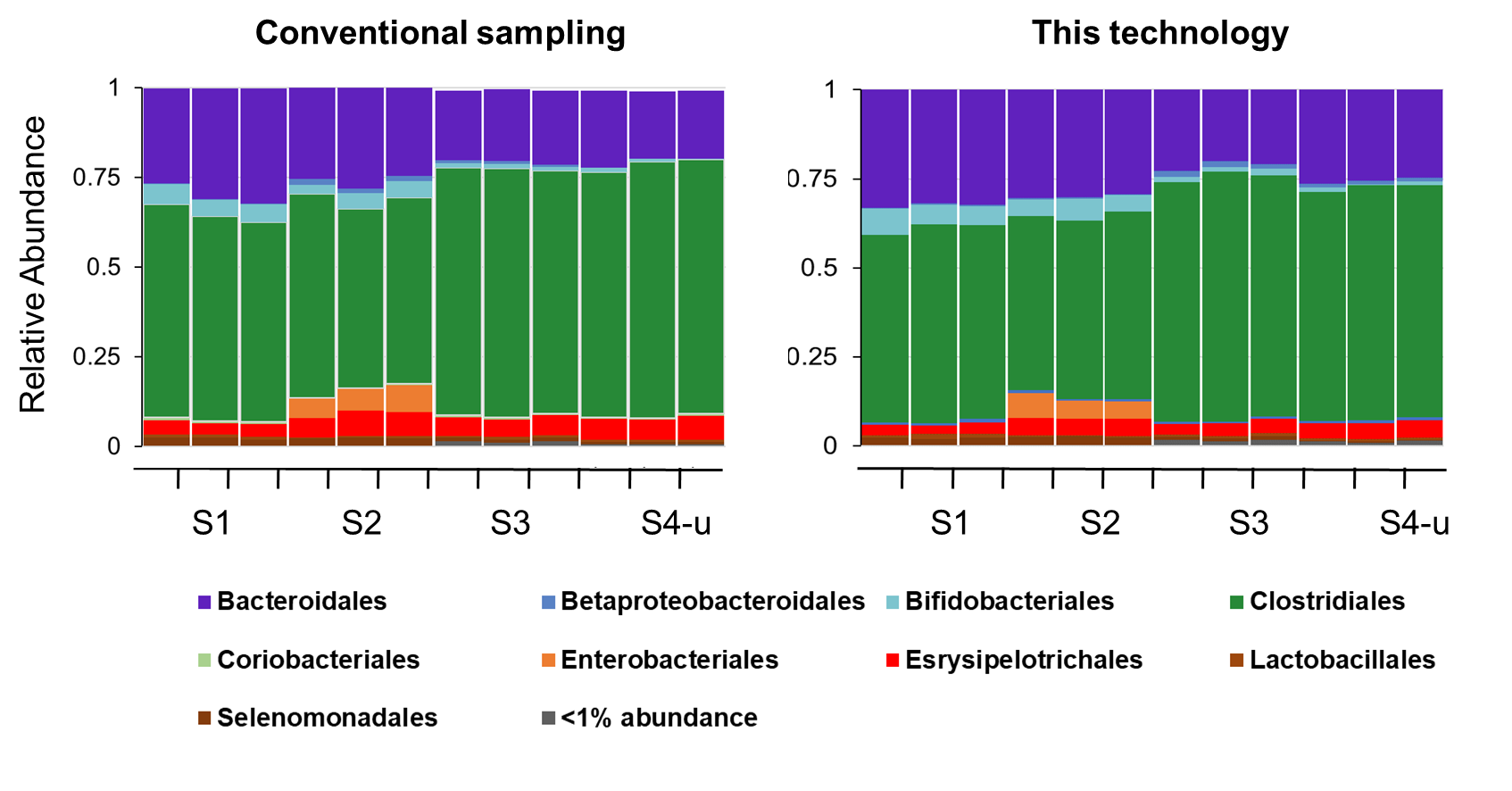
**

**Figure S5 Turbidity sensor calibration.** SEN085 analog turbidity sensor output voltage change and turbidity meter (Hach) 2100Q for solutions of feces surrogate in a range of solid content. The read-out are linear in the range 0-1000 NTU of the turbidity meter as well as up to 30 mg/ml.

**Figure S6** Pictures of the turbidity sensor SEN085 and its installation in the toilet rear exit. A. analogue turbidity sensor as purchased. B The turbidity probe installed in the rear exit of a toilet. C the turbidity adapter is secured outside the toilet effluent pipe and connected to an Arduino for data recording.

**
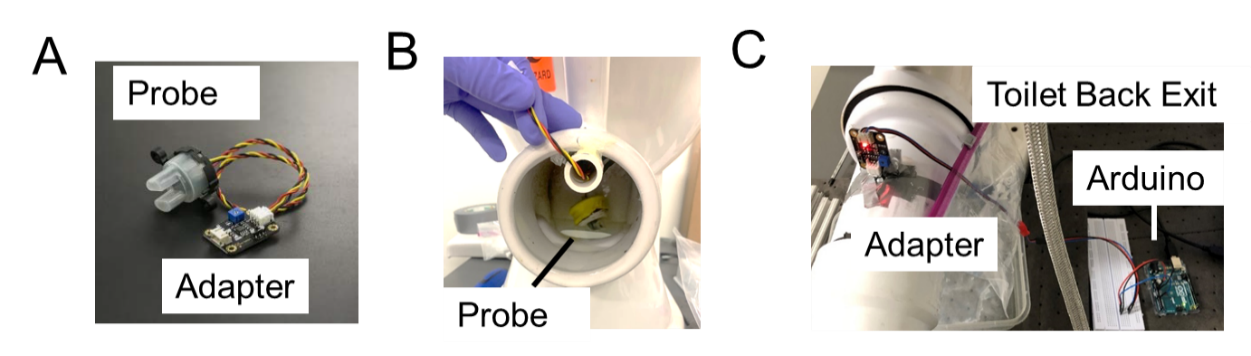
**
